# The role of saline contrast echocardiography in imaging partial coronary sinus septal defect

**DOI:** 10.1186/s44348-026-00072-x

**Published:** 2026-05-27

**Authors:** P Harish, Deepa Sasikumar, Ahmed Wayez, Jineesh Valakkada

**Affiliations:** 1 Department of Cardiology, Sree Chitra Tirunal Institute for Medical Sciences and Technology, Trivandrum, India; 2 Department of Imaging Sciences and Interventional Radiology, Sree Chitra Tirunal Institute for Medical Sciences and Technology, Trivandrum, India

**Keywords:** Coronary sinus septal defect, Saline contrast echocardiography, Left superior venacava

A 12-year-old girl was evaluated for asymptomatic cardiac murmur. Electrocardiogram showed incomplete right bundle branch block and chest x-ray demonstrated cardiomegaly with increased pulmonary blood flow. Transthoracic echocardiography (TTE) showed dilated right atrium (RA) and right ventricle (RV) with intact interatrial septum (Fig. 1A). Coronary sinus (CS) was dilated and there was persistent left superior vena cava (LSVC). As partial CS septal defects are a common cause of occult pre-tricuspid left-to-right shunts, saline bubble contrast echocardiography was done through the left cephalic vein. Bubbles immediately appeared in CS, RA, RV and left ventricle (LV), without significant opacification of the left atrium (LA) (Fig. 1B, C, Video S1). A closer inspection of the subcostal short axis view showed a defect in the middle part of the CS with shunt from LA to CS (Fig. 1D). Diagnosis of type III CS septal defect [1] was confirmed on cardiac computed tomography (CT) (Fig. 2) and patient underwent surgical closure.

**Fig. 1:**
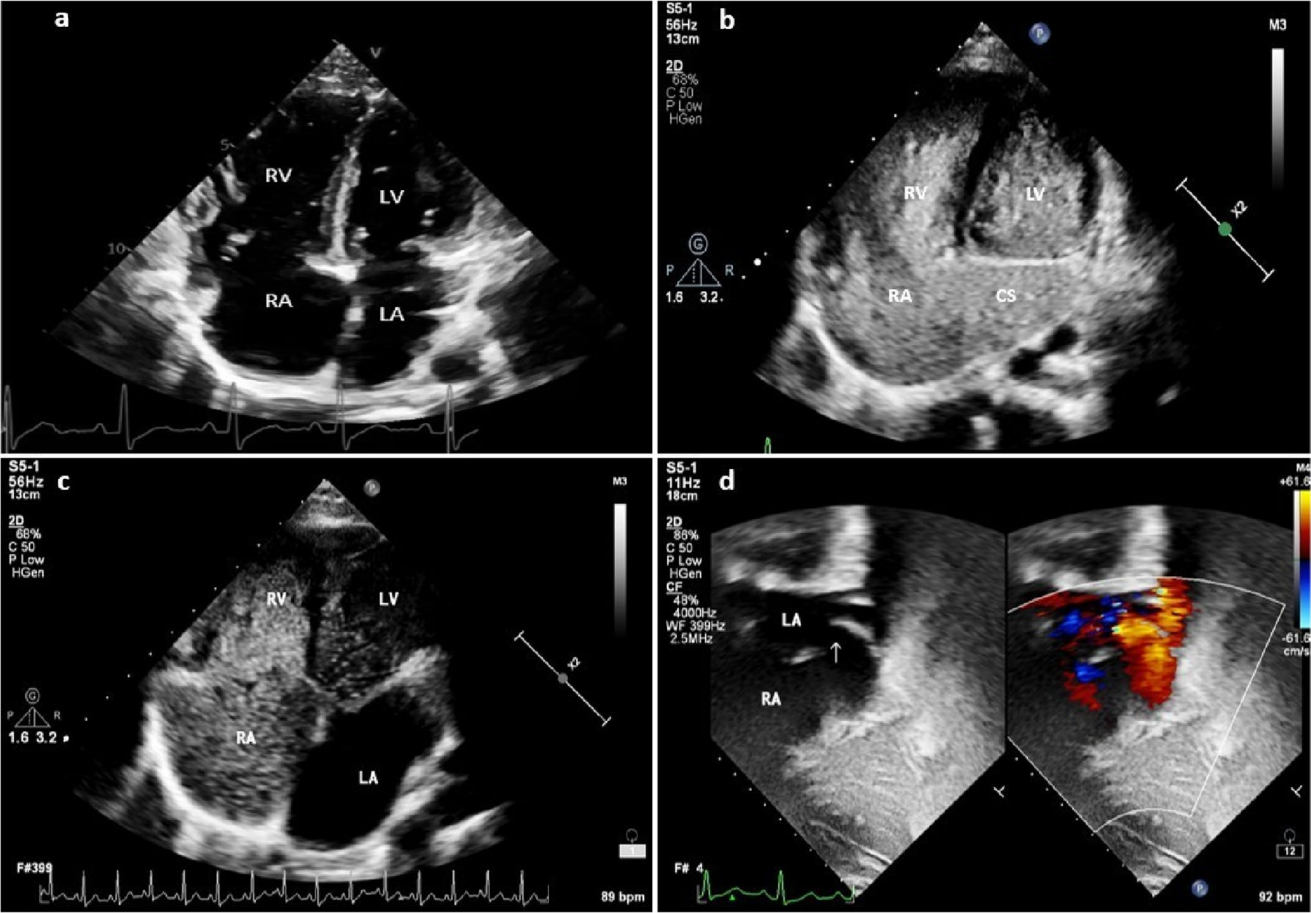
Apical four-chamber views and subcostal sagittal view. **A** Apical four-chamber view demonstrating dilated right atrium (RA) and right ventricle (RV). **B** Apical four-chamber view after contrast injection through left antecubital vein demonstrating the appearance of contrast bubbles in coronary sinus (CS), left ventricle (LV), RA, and RV. **C** Apical four-chamber view demonstrating the appearance of contrast bubbles in LV, RA, and RV with no apparent contrast opacification in left atrium (LA). **D** Subcostal sagittal view demonstrating dilated CS and defect in the roof of CS (arrow). Color flow doppler (color comparison on the right) shows a red jet from LA to CS to RA

**Fig. 2:**
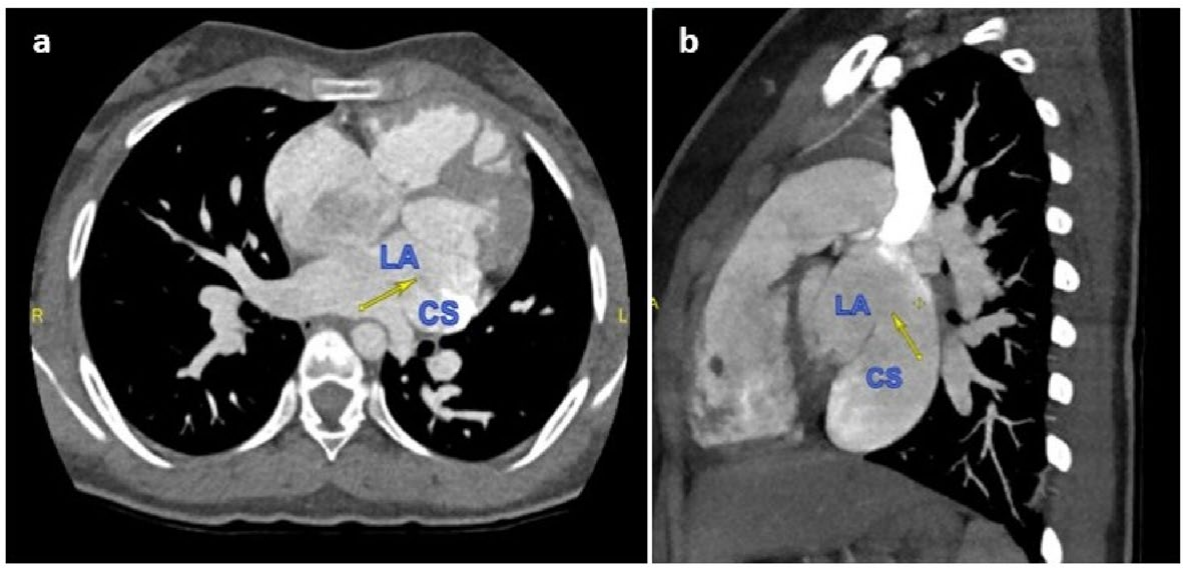
Cardiac computed tomography (**A**) axial and (**B**) oblique sagittal views demonstrating dilated coronary sinus (CS) and defect in the posterior wall of left atrium (LA) communicating with CS (arrows)

CS septal defects in isolation are rare congenital cardiac defects characterized by a tissue deficiency separating the CS from LA due to hypoplasia of left atriovenous fold during embryonic development [2]. Kirklin and Barratt-Boyes [1] classified CS septal defects as follows: type I, completely unroofed CS with LSVC; type II, completely unroofed CS without LSVC; type III, partially unroofed midportion; and type IV, partially unroofed terminal portion. CS septal defect is commonly associated with persistent LSVC. Partial unroofing of CS results in a predominantly left-to-right shunt through the CS (LA → CS → RA), but a transient right-to-left shunt into the LA may occur especially with a Valsalva maneuver. It is often difficult to identify these posterior defects by TTE. Color flow doppler are sometimes unable to demonstrate the defect due to a poor window or other technical factors. Dilatation of CS is not always diagnostic of a CS septal defect especially in the presence of persistent LSVC. Complete unroofing of CS with persistent LSVC is easily demonstrated by bubble saline contrast injected into the right or left arm, which results in immediate opacification of LA, LV, RA, and RV (Fig. 3, Video S2).

**Fig. 3:**
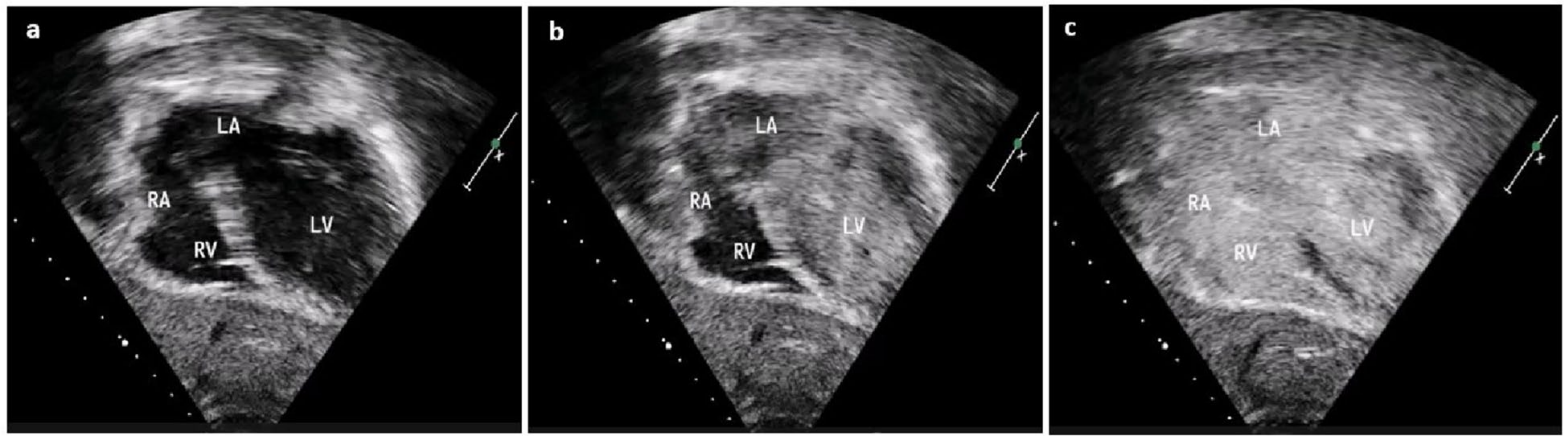
(A–C) Saline contrast echocardiography with series of images in a different patient. Subcostal four-chamber view after left arm contrast injection, demonstrating contrast bubbles filling left atrium (LA), left ventricle (LV), right atrium (RA), and right ventricle (RV), suggestive of completely unroofed coronary sinus with persistent left superior vena cava

In partially unroofed CS with defect in the terminal portion, bubbles will appear immediately in RA, RV and also in the LA, whereas in those with defect in the mid portion with persistent LSVC (as in our case), bubble contrast has been demonstrated to appear in RA, RV, and LV [3]. The reason for the appearance in LV without much evident filling of LA is not clear and has been attributed to the proximity of the mitral valve to the CS, which may have caused the bubbles to escape rapidly from LA to LV. A literature review by another author noted that in partially unroofed CS, contrast bubbles may enter the LV directly from the CS [4]. In complete or partial unroofed CS without LSVC, the bubble contrast injected into the right or left arm will not reach the CS as it drains only the cardiac veins and it is not useful in this setting.

CS septal defects are rare causes of pre-tricuspid left-to-right shunts. Such defects, especially partial ones, are often missed on transthoracic echocardiography since they are deep and posterior defects. Dilatation of CS is an important diagnostic clue to suspect these defects. Persistent LSVC often accompanies these defects and hence dilated CS cannot be solely attributed to CS septal defects. Complete and partial unroofing of CS in the presence of LSVC is easily demonstrated by saline contrast echocardiography. Less well known is the utility of this modality in detecting unroofing of the CS in the mid portion where the saline appears first in LV. Knowledge of this pattern of appearance of saline contrast first in the LV in this setting will empower the clinician to make a diagnosis of this entity without additional imaging such as cardiac CT.

Diagnosis of partially unroofed CS by transesophageal echocardiography (TEE) is difficult as these defects are located posteroinferiorly. Identification of CS itself can be challenging and dilated CS can often be mistaken for inferior vena cava on midesophageal bicaval view. The role of TEE in these cases is thus limited to intraoperative assessment of surgical success. In patients with unroofed CS associated with a persistent LSVC, saline contrast echocardiography is a useful noninvasive diagnostic modality. However, in the absence of LSVC, its diagnostic utility is limited and cross-sectional imaging with cardiac CT or magnetic resonance imaging (MRI) provides better anatomical delineation. CT and MRI enable precise characterization of the size and extent of the CS roof defect, its relationship to the CS ostium and LA wall, proximity to the mitral valve, the course of a persistent LSVC, and the presence of associated cardiac anomalies. Therefore, a multimodality imaging approach is often essential for accurate diagnosis and comprehensive evaluation of these defects.

## Supplementary Information

Additional file 1: Video S1. Apical four-chamber view after left arm contrast injection demonstrating the appearance of contrast bubbles in left ventricle (LV), right atrium, and right ventricle. There is no filling of contrast in left atrium as expected in partial unroofed coronary sinus (CS) but the appearance of contrast bubbles in LV here is probably due to the close proximity of the CS defect to the mitral valve.

Additional file 2: Video S2. Subcostal four-chamber view with left arm contrast injection demonstrating contrast bubbles in the left atrium and left ventricle filling through coronary sinus (CS) followed by right atrium and right ventricle, suggestive of completely unroofed CS with persistent LSVC (type I CS atrial septal defect).

## Data Availability

Not applicable.

## Abbreviations

CS: Coronary sinus
CT: Computed tomography
LA: Left atrium
LSVC: Left superior vena cava
LV: Left ventricle
MRI: Magnetic resonance imaging
RA: Right atrium
RV: Right ventricle
TEE: Transesophageal echocardiography
TTE: Transthoracic echocardiography
